# A new bio imagery user-friendly tool for automatic morphometry measurement on muscle cell cultures and histological sections

**DOI:** 10.1038/s41598-024-53658-0

**Published:** 2024-02-07

**Authors:** Aurélien Brun, Guillaume Mougeot, Philippe Denis, Marie Laure Collin, Pierre Pouchin, Christophe Montaurier, Stéphane Walrand, Frédéric Capel, Marine Gueugneau

**Affiliations:** 1 UMR1019 Unité de Nutrition Humaine (UNH), INRAE, Université Clermont Auvergne, Clermont-Ferrand, France; 2 iGReD CNRS, INSERM Université Clermont Auvergne, Clermont-Ferrand, France

**Keywords:** Biological techniques, Cell biology, Physiology

## Abstract

TRUEFAD (TRUE Fiber Atrophy Distinction) is a bioimagery user-friendly tool developed to allow consistent and automatic measurement of myotube diameter in vitro, muscle fiber size and type using rodents and human muscle biopsies. This TRUEFAD package was set up to standardize and dynamize muscle research via easy-to-obtain images run on an open-source plugin for FIJI. We showed here both the robustness and the performance of our pipelines to correctly segment muscle cells and fibers. We evaluated our pipeline on real experiment image sets and showed consistent reliability across images and conditions. TRUEFAD development makes possible systematical and rapid screening of substances impacting muscle morphology for helping scientists focus on their hypothesis rather than image analysis.

## Introduction

Lean body mass, in particular muscle mass, is an excellent predictive survival factor in many diseases^1–3^. Skeletal muscle is a heterogenous tissue containing fibers with different morphological, functional, and metabolic characteristics^4^. Myofibers express different myosin isoforms that are related to specific properties of muscle fiber contraction. Two classes of muscle fibers can be distinguished in humans: slow-type oxidative fibers, which are more resistant to fatigue, consume more oxygen, and express type I myosin; and fast-type glycolytic fibers, which generate more force, express type II myosin (Type IIA and IIX)^5,6^. In rodents, type I fibers are similar to human but they have 3 different types of fast-glycolytic fibers expressing type II myosin (Type IIA, IIB and IIX). Environmental changes, aging^7^, drugs, or diseases (like cachexia^8^ or metabolic disorders^9^) can affect skeletal muscle mass, fiber composition and size, resulting in perturbations of skeletal muscle functions. On the contrary, exercise or steroids could induce skeletal muscle hypertrophy^10,11^. Thus, to study muscle function and/or to prevent muscle atrophy, researchers use various models ranging from cell cultures to tissue biopsies from rodents to humans. However, there is no modulable, efficient, and reliable pipeline to quantify image morphometry captured using these models because several challenging issues remain to be solved. Atrophy and hypertrophy are assessed by measuring myotube diameters on cells in culture^12–15^ or skeletal muscle fiber cross-sectional area (CSA) from tissue biopsies^16,17^. Fiber typing, which aims to establish the proportion of each fiber type, is also performed using immunofluorescence staining. However, most of these experiments are time-consuming, and while semi-automatic and automatic tools^18–20^ have been developed over the years, most quantification is still performed manually using open-source Fiji (Fiji is just ImageJ) platform^21^. Furthermore, researchers using phase contrast microscopy to analyze images from myotubes in culture might have figured out the high variability of contrast between microscopes, image batches, and even in different fields from the same sample. Indeed, contrast variability associated with a low contrast between cells and background compromises the selection of a common threshold to segment highly confluent, adherent cells in a heterogenous batch of images^22^. While this is a well-known problem, recent advances in bio-imagery using deep learning support (as Unet^23,24^, Cellpose^25^, or Ilastik^26^) have provided new perspectives in this field^27,28^. Nevertheless, efficient segmentation remains challenging for some cell types, such as myoblasts differentiated into myotubes (even on Omnipose^29^) as size and shape may be variable between myotubes from the same field. Consequently, it was necessary to solve an over/under-segmentation issue and prevent the consideration of myotube nodes for the estimation of cell diameter.

In this study, we have developed an easy-going image analysis pipeline allowing a consistent and automatic measurement of myotube diameter in vitro and muscle fiber type and area of rodents and human muscle biopsies. The in vitro tool, named TRUEFAD Cells, can be used under the FIJI-ImageJ software distribution with only phase-contrast microscopy images as an input allowing the analysis of a batch of images without any sample preparation. For muscle histology, the tool that we have developed, named TRUEFAD Histo, uses images coming from the acquisition of immunofluorescent staining. It can perform a segmentation of skeletal muscle fibers and a measurement of fiber CSA. In addition, it quantifies up to three different myosins for establishing the fiber typing, or other parameters depending on the antibodies used or the research question. It is completely automated and does not require the use of high-quality images. To our knowledge, TRUEFAD is the first automatic tool able to measure the diameter of differentiated myotubes in vitro and in vivo fiber typing. In addition, the speed of this fully automated method significantly increases the number of fibers or myotubes analyzed and, therefore, improves the statistical power of the analyses.

## Results

### Analysis of myotube diameters using TRUEFAD cells

After numerous preliminary attempts of conventional segmentation techniques (K-means clustering, Watershed, Active contour), approaches for fiber granulometry^30^ and machine learning/deep learning usage, we addressed this issue by training our own semantic segmentation deep learning algorithm to detect myotube’s structures on any type of phase contrast images.

**Figure 1:**
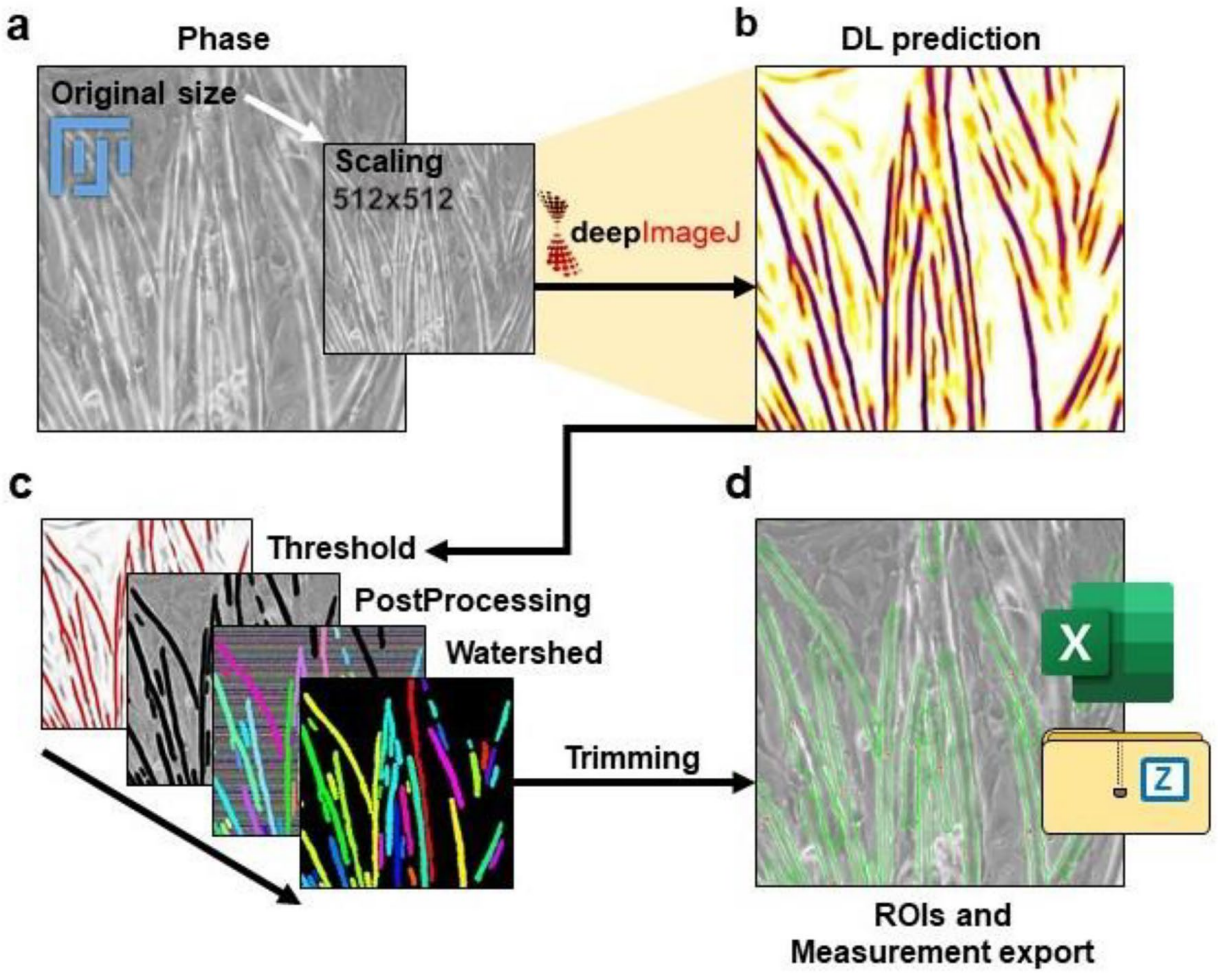
TRUEFAD Cells workflow. (**a**), Scaling image to 512 × 512 pixels from native 8-bit phase contrast images. (**b**), Deep Learning (DL) prediction obtained with DeepImageJ from the scaling image. (**c**), Post-processing steps apply to the DL prediction image. (**d**), ROIs and measurement export obtained at the end of the post-processing step.

All the TRUEFAD Cells workflow was designed to run on FIJI distribution with few dependencies that need to be installed (Fig. 1). Each image follows the same pipeline. The image is firstly scaled down (Fig. 1a) to be run with our deep learning (DL) model thanks to DeepImageJ^31^ (Fig. 1b). We obtain a transformed image that corresponds to a probability map of all myotube’s center. This image could be used in the post-processing steps (directional and morphological filtering) to obtain a frame, applied to the original phase contrast image. A top-hat filter is run on each putative myotube from the original image (Fig. 1c) to remove artifacts and enhance the contrast with the cell border. All other structures present in the original images are then covered with a high noise filter, preventing wrong, false-positive acquisitions in the following steps. Marker-controlled watershed segmentation is then run on identified myotubes according to the step-by-step MorphoLibJ “Impose extended local minima” function^32^.

Labels obtained from the segmentation process correspond to the myotubes detected on the image and eventual artifacts. Undesired labeled objects could be removed by label size filtering according to user preference parameters. Putative myotubes are then trimmed by their geodesic elongation also using user-defined criteria. Each myotube is then extracted from the image and label holes are filled following interpolation. The area and orientation are saved. Nine measures of diameter are then obtained, perpendicularly to the main axis of the myotube.

Measures from all myotubes on each image are exported in a spreadsheet on computer’s desktop as well as the mean diameter, global area, and orientation. Each label is saved as ROI that is exported as well as the overlay of the original image (Fig. 1d).

**Figure 2:**
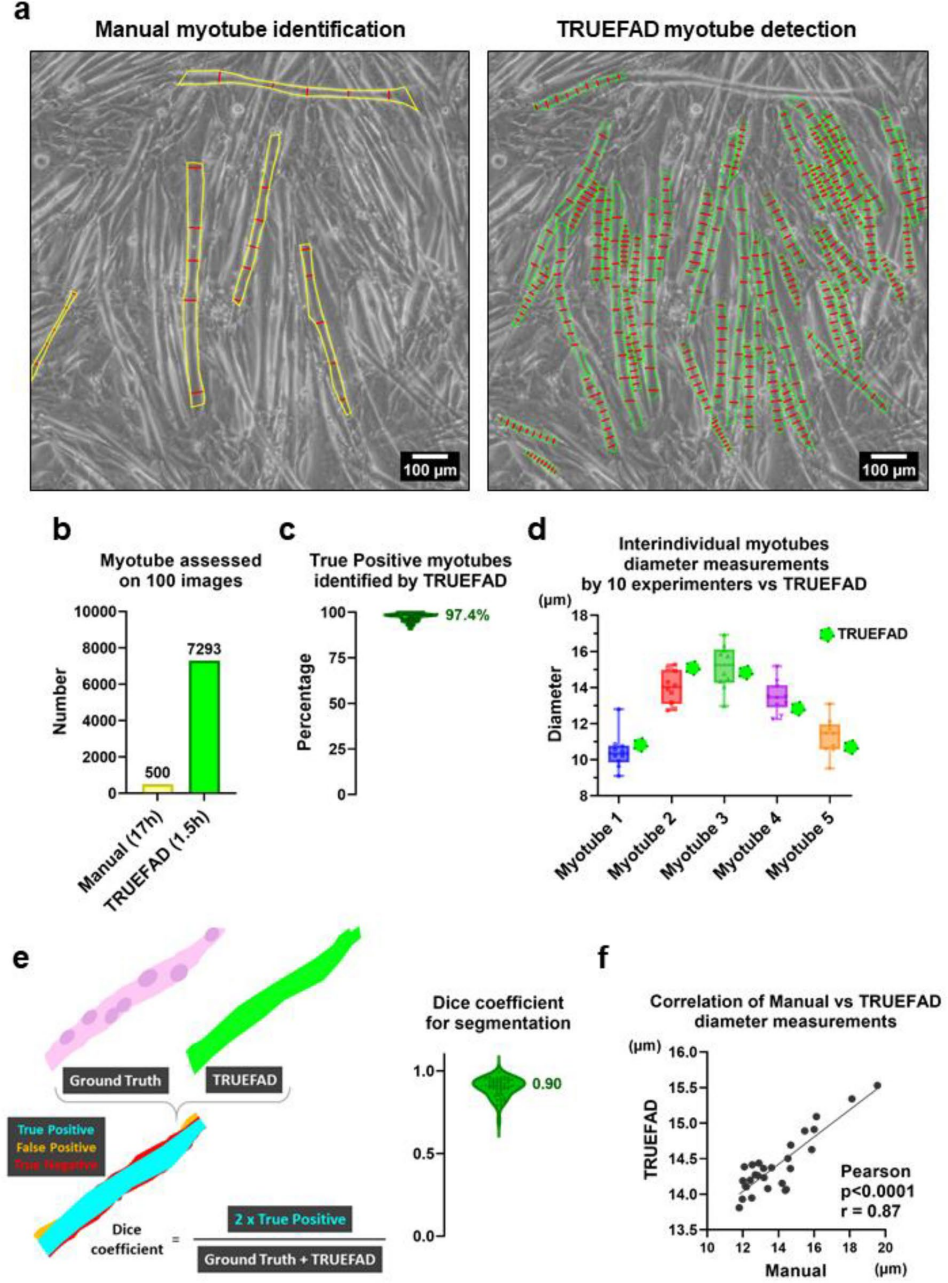
TRUEFAD Cells metrics. (**a**), Five manually measured diameters commonly done on five C2C12 myotubes per image (left panel) compare to nine systematically made diameters by TRUEFAD Cells on each detected myotube. (**b**), Number of myotubes detected by TRUEFAD Cells on 100 images or following a conventional manual analysis. (**c**), Percentage of correct myotube’s identification by TRUEFAD Cells (Violin plot with median, *n* = 7293 myotubes assessed on 100 Images). (**d**), Variability in mean diameter measurement across 10 experimenters for five selected myotubes compared to TRUEFAD Cells, the center line of boxplot indicates the median value. (**e**), Assessment of Dice coefficient quality of TRUEFAD Cells segmentation versus Ground Truth (Violin plot with median, *n* = *50* myotubes). (**f**), Correlation between mean manual and TRUEFAD diameters measurements on images from 28 experiments.

Although the conventional manual analysis consists of five measures made on five myotubes per image subjectively chosen, TRUEFAD performs an analysis on all the myotubes detected on the image with 9 measures for each (Fig. 2a). Adaptive capabilities of our pipeline using different image’s qualities for myotube prediction are available in Extended Fig. 1.

The performance of TRUEFAD Cells to identify myotubes was compared to manual processing (Fig. 2b). Out of a batch of 100 images of differentiated C2C12 cells, 7293 myotubes were detected and measured by TRUEFAD in 90 min in comparison to 500 myotubes which required around 17 h to be measured by hand. Correct identification, segmentation and measures were obtained for 97.4% (*SEM* ± *0.22*) of the myotubes identified by TRUEFAD Cells (Fig. 2c). To assess the robustness of automatic measurement in comparison to manual measurement, 10 experimenters with a significant background in the use of C2C12 myotubes have individually measured five times the same batch of 5 myotubes. We performed a comparison between the mean interindividual measurement of the myotubes and the diameter evaluation made by TRUEFAD Cells (Fig. 2d). Results showed no difference between the manually and TRUEFAD quantifications. In order to evaluate the robustness of systematic segmentation of the myotubes in comparison to ground truth manual segmentation, we randomly selected one myotube detected by TRUEFAD Cells on each image from the same batch of 50 images and evaluated the Dice coefficient (Fig. 2e). The dice coefficient was 0.9 ± 0.008 reflecting high confidence in the border detection. The mean myotube diameters computed by TRUEFAD Cells were very similar to those obtained manually (Fig. 2f) as a robust correlation was found between the two approaches (*Pearson p* < *0.0001; r* = *0.8752; n* = *28 mean diameter from experiments*).

**Figure 3:**
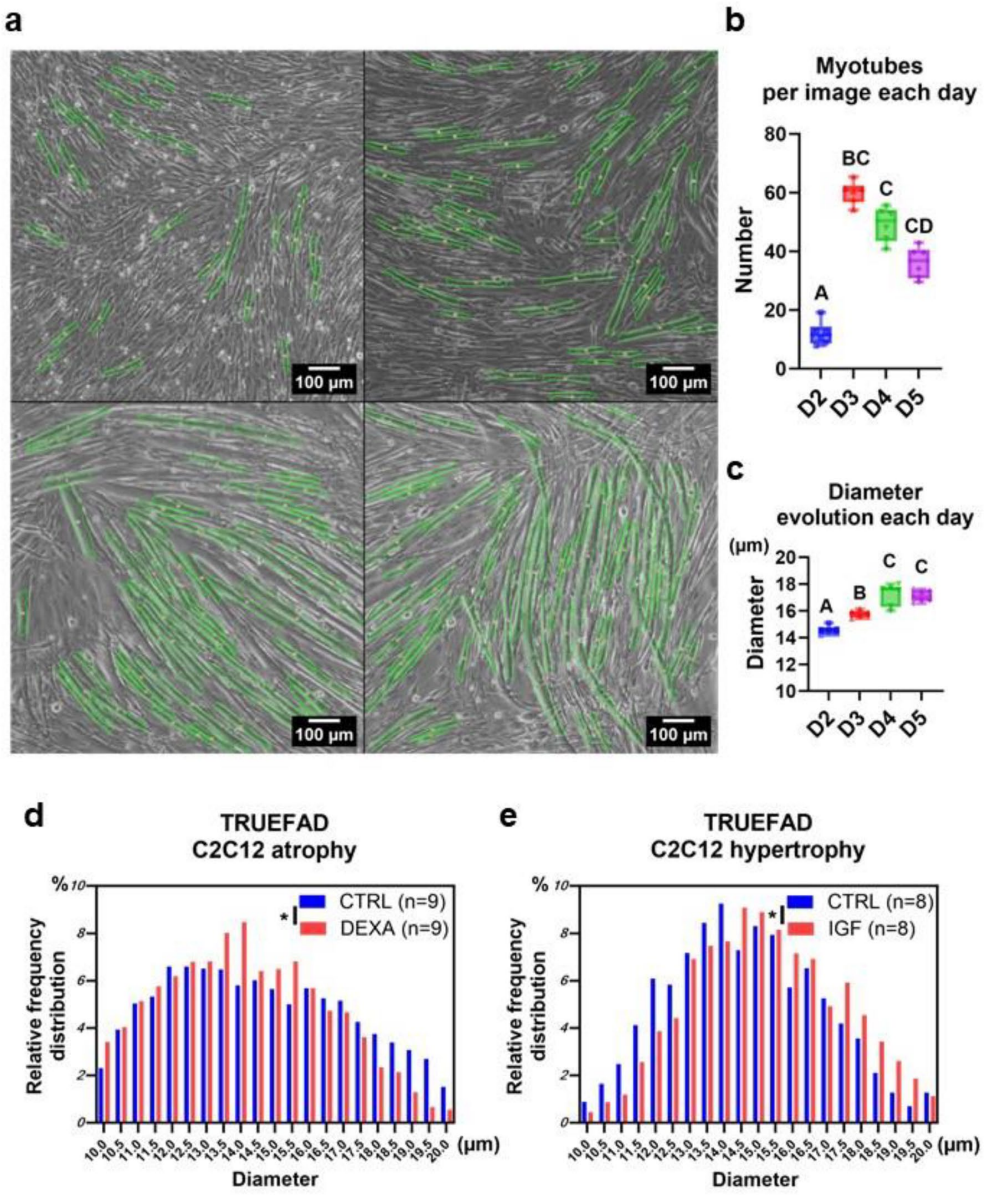
Application of TRUEFAD Cells to study myotube morphology during differentiation and upon exposure to IGF-1 or dexamethasone. (**a**), Myotubes detected by TRUEFAD Cells during differentiation of C2C12 cells (*n* = 10 images per day on *n* = 6 technical replicates). (**b**), Number of detected and (**c**), myotubes mean myotube diameter at day 2 (D2), 3 (D3), 4 (D4) and 5 (D5) of cell differentiation. The centerline of the boxplot indicates the median value (*n* = 6 replicates). (**d**), Heatmap representation of the distribution of C2C12 myotube diameters after exposure to Dexamethasone (DEXA) at 10^−6^ M for 48 h (*s.e.m.* ± 0.12, *n* = 9 independent experiments on a total of 2998 and 3559 myotubes in CTRL and DEXA condition respectively, ***p < 0.001). (**e**), Heatmap representation of the distribution of C2C12 myotube diameters after treatment with IGF1 at 50 nM for 48 h (*s.e.m.* ± *0.12, n* = 8 independent experiments on a total of *1610* and *1646* myotubes in CTRL and IGF1 condition respectively, *p < *0.05*). One-way ANOVA with multiple comparisons was performed in (**b**) and (**c**), with statistical differences identified with different letters. Paired student t test was performed in (**d**) and (**e**).

We next challenged TRUEFAD Cells on C2C12 images obtained throughout myotube differentiation from day two to five (Fig. 3a). As previously described in the literature, myotubes formation from myoblasts starts after 2 days of differentiation^33^. Then, newly formed myotubes fuse to form larger myotubes and their number decreases. The mean numbers of detected myotubes per image which ranged from 11.92 ± *1.65* at D2, 60.03 ± *1.58* at D3, 49.20 ± *2.38* at D4 to 36.18 ± *2.17* at D5 (Fig. 3b, n  = *6 technical replicates*). Myotube diameter (Fig. 3c) significantly differed between D2 (14.56 ± *0.18* µm), D3 (15.75 ± *0.18* µm*)* and D4 & D5 (17.21 ± *0.18* µm*;17.09* ± *0.18* µm*)*. This result shows TRUEFAD is able to robustly follow the differentiation of C2C12 myotubes.

We further explored if changes in myotube diameters could be detected by TRUEFAD Cells. Myotubes atrophy or hypertrophy was induced respectively by dexamethasone (DEXA, 10^-6^ M) for 48 h and IGF-1 (50 nM) for 72 h. Results show that TRUEFAD Cells quantification could detect a decrease of myotube diameter induced by DEXA compared to the control condition (13.98 µm vs. 14.67 µm, *p* < 0.001) as previously described^34^ (Fig. 3d). IGF-1 induced an increase in  the mean diameter compared to the control condition (*15.02 µm vs.14.50 µm; p* < *0.05*) as previously observed^35^ (Fig. 3e). As illustrated on Extended Fig. 2, TRUEFAD Cells was also able to detect the atrophy of human myotubes after 48 h of treatment with DEXA. For this last analysis, only three biological samples corresponding to a total of 15 images per condition were enough to unravel significant myotube atrophy (*p* < 0.05).

### Analysis of muscle fiber morphology and typology using TRUEFAD Histo

**Figure 4:**
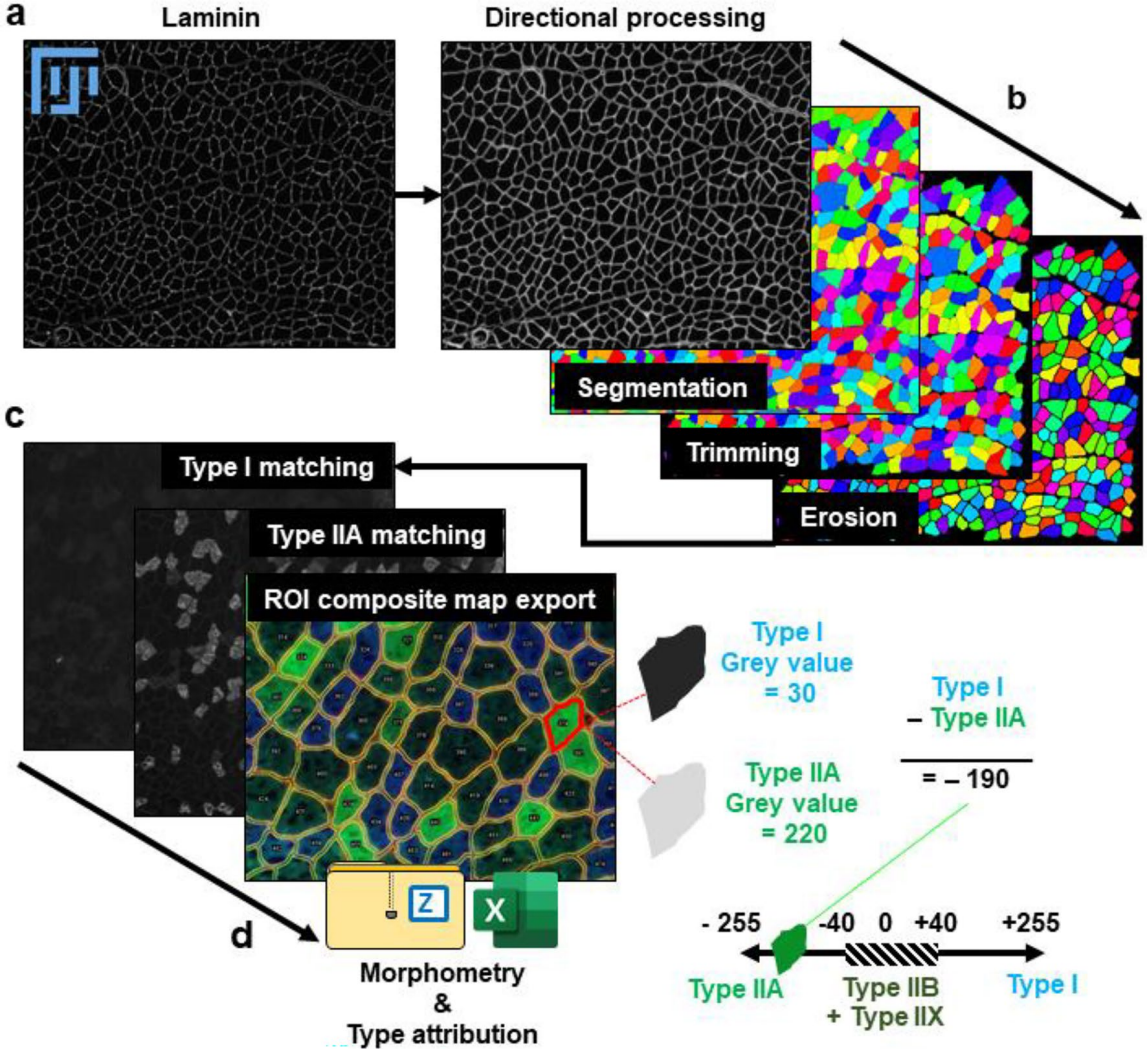
Workflow of TRUEFAD Histo. (**a**) Representative laminin immunostaining image. (**b**), Laminin segmentation processing using directional filters followed by local contrast adjustment and marker-imposed watershed segmentation. (**c**), Representatives images of fiber type I and IIA immunostaining and the ROI composite map export generated by TRUEFAD histo. (**d**), Quantification of gray value intensity for each fiber on each immunostaining images and fiber type classification (Type I, Type IIA and Type IIB + IIX).

As TRUEFAD was able to identify myotubes and estimate morphological quantitative data, the same analysis strategy and a similar pipeline were applied to images from muscle histological experiments. Thus, the TRUEFAD Histo plugin was created to improve fiber segmentation and facilitate typological characterization of fibers using immunohistological imaging. TRUEFAD Histo was developed in ImageJ macro language to ensure up-to-date follow-up of the service and easy-to-use configuration. A “Help” button on the graphical user interface links to online supportive information and a user guide. Instead of a conventional threshold-based approach, TRUEFAD Histo (Fig. 4) proposes a segmentation based on the immunodetection of laminin using directional filters followed by local contrast adjustment and marker-imposed watershed segmentation (Fig. 4b). Labels obtained from the segmentation process correspond to muscle fibers and noisy structures detected in the image. Removal of artifacts can be done using a label size filtering chosen by the user followed by a geodesic elongation trimming. If desired, manual checking can be done at this step.

In order to classify fibers according to the metabolic type (I, IIA and IIB + IIX), type I and type IIA fibers were immunodetected using 2 different fluorescent probes.

A label mask extracted from the segmentation processing (Fig. 4b) is applied to each of the two immunolabeling per section (Fig. 4c), allowing the quantification of the gray value for each fiber in both channels (Type I and IIA respectively, Fig. 4d). Identification of the metabolic type is determined by subtracting for each fiber the mean gray value of the type I signal, minus the mean gray value of the type IIA signal. A resulting value below − 40 indicates a pure type IIA signal. Values higher than + 40, indicate a pure type I signal and values between − 40 and + 40 are indicative of type IIB and IIX fibers. Data related to the area, perimeter, minimal “Feret's diameter”, type I, type IIA gray value, and type probability attribution, including putative type I/IIA hybrid fibers, is exported as a spreadsheet on the computer’s desktop. The confirmation of hybrid fibers should be done manually as variability in signal intensity and background implies that it could not be automatically achieved. TRUEFAD Histo allows the user to run all of these steps in an automated pipeline (n°3: See the methods section) to analyze hundreds or thousands of images from the same experiment. TRUEFAD Histo proposes several other pipelines, such as n°1: Import and work on label Images, n°2: Simple segmentation of laminin images, and n°4: Grey value measures for 3 fluorescent images. This last pipeline allows the automatic quantification of signal from immunohistological detection of type I, IIA and IIX fibers. The user could then be able to use the the excel output to manually identify each type according to the setting of thresholds that could vary between experiments.

**Figure 5:**
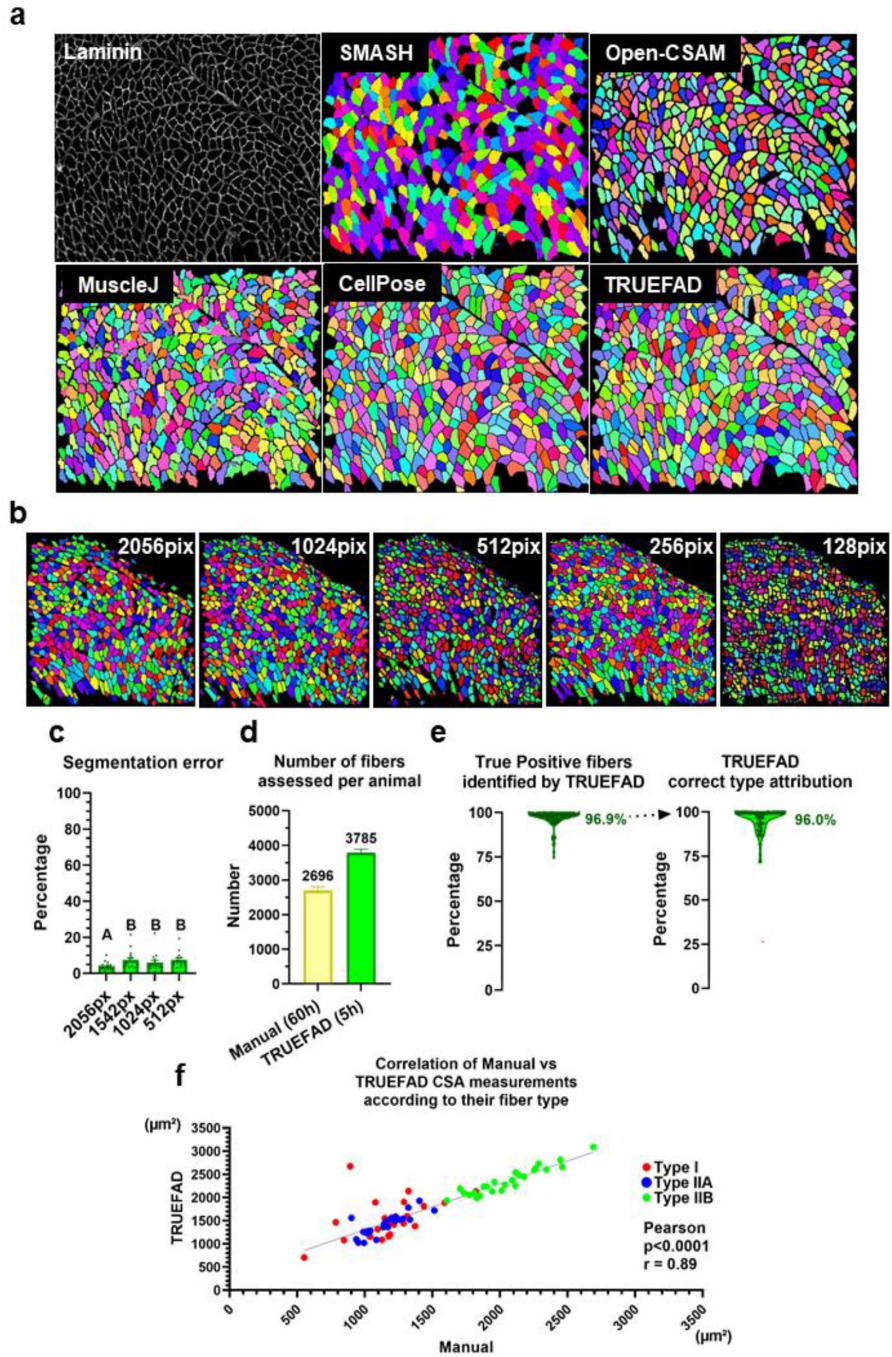
TRUEFAD Histo reliability. (**a**) Representative results of laminin segmentation from SMASH, OpenCSAM, Muscle J, Cellpose, and TRUEFAD tools from the same raw original image. (**b**) TRUEFAD Histo reliability in scale-down images. (**c**) Percentage of fibers incorrectly segmented according to image resolution (*n* = 15 images). (**d**) Number of fibers identified by TRUEFAD Histo and manually-supervised open-CSAM. (**e**) Quantification of TRUEFAD Histo segmentation errors on 117 images (n = 25 muscles for a total of 50,483 fibers, left panel) and percentage of correct assignment of fiber type among the 48,918 identified fibers (right panel). (**f**) Correlation between manually supervised open-CSAM cross-sectional area (CSA) measurement according to type and TRUEFAD results. Error bars in (**d**) and (**e**), are s.e.m. One-way ANOVA with multiple comparisons were performed on resolution data shown as boxplots in c, with different letters indicating statistical differences. Pearson correlation test in (**f**) was calculated with each point corresponding to a single individual (sum of data from 5 to 7 different fields).

TRUEFAD Histo laminin segmentation of muscle fibers competed with up-to-date CellPose segmentation without any issues related to deep learning in term of interoperability and resource consumption (Fig. 5). Downscaling images reduced their processing time from 58.3 s with 2056 × 2056 pixels to 2.8 s with 512 × 512 pixels (Fig. 5b, see Methods section for full benchmark test). This data reduction increased the amount of segmentation error and reduced the precision of the segmentation (Fig. 5c) 1.0X (*4.0%* ± *0.6*), 0.75X (*7.4%* ± *1.3*) 0.5X (*6.0%* ± *1.4*) 0.25X (*7.5%* ± *1.1)*. All subsequent analyses were made on native 2564 × 2056 images. The number of fibers detected by TRUEFAD Histo was 29% higher than fibers manually verified after Open-CSAM^36^ detection (Fig. 5d) with segmentation error restricted to only 3.1% (Fig. 5e, left panel). Even low-quality images were able to be analyzed thanks to the morphological and directional filtering steps that closed cell bordered and cleaned artifacts in the center of the cells. Fiber type attribution processed by TRUEFAD Histo on a sample of 48 918 fibers led to 96.0% of correct attribution (Fig. 5e, right panel). Out of the 117 images used for this validation analysis, a Pearson correlation test of the cross-sectional area (CSA) was made for each of the three fiber types (Fig. 5f) and showed a strong positive correlation (*Pearson p* < *0.0001; r* = *0.8960; n* = *25 rats with CSA of respectively 3 fiber types assessed*) between the CSA evaluated by a classical manual process in comparison to TRUEFAD Histo. A similar positive correlation was obtained without consideration of fiber type (Extended Fig. 3).

## Discussion

This article introduced the TRUEFAD package as a new powerful and robust bio-imagery tool for all research activities related to muscle health. The package has been designed with deep concern about it being user-friendly, open source, and highly adaptive to ensure that everyone could use it on the open-source Fiji software. We are confident that these two tools will bring high interest to the scientific community working on skeletal muscle. Thanks to the online resources and tutorial, we hope that this software will boost the research about fundamental and pathological aspects of muscle biology.

TRUEFAD Cells required the training of a deep learning model to ensure the analysis of a heterogeneous batch of images due to variability in the quality of phase contrast acquisition. It solved the troubles related to the identification of a common threshold for myotubes segmentation due to differences in size, shape and variability in segmentation choices between users. For histological analysis, we decided to develop a pipeline that did not depend on deep learning training for histological sections but which remains highly customizable in many ways (Type I and IIA fibers could be replaced with other fluorescent immunostaining). The processes that we used was based on the immunodetection of laminin for fiber identification but any other markers that outline individual fibers could be used. It overcome noise-related issues and signal heterogeneities among images^20^ and was much better than other available tools and as efficient as the recently developed CellPose pipeline^25^ all in one open-source place. Results were validated by comparing TRUEFAD outputs to robust and multi-user analyses of the same batches of images, showing that the pipeline could be immediately used by researchers interested in muscle health.

TRUEFAD Cells and Histo was developed to overcome the gap between the actual high time-consuming analysis of images from the histological section or cell culture images and the need for screening of potentially harmful or beneficial compounds that may respectively impede or protect muscle morphology. Both pipelines were set up to be used in any lab to produce a high amount of data that had never been achieved by any alternatives. While numerous laboratories and companies have developed solutions during the last decade for the assessment of muscle fiber’s area, only a few examples propose fiber typing. With this new tool, we aim for better standardization of the practice for now and for a major improvement of reproducibility in the future.

TRUEFAD has therefore some potential limitations. For example, the detection of hybrid fibers expressing two myosin heavy chain isoforms requires manual analysis of raw signal intensities from myosin immunolabeling that are exported in the excel file. These fibers are considered transitional between the respective pure fiber types and and despite their importance in some pathological conditions, the expression of the different myosin isoforms could be heterogeneous along the fiber^37^. Consequently, a methodology based on cross section is probably not the most relevant method for their identification. Considering our main goal, that was to identify metabolic shifts between different conditions, and the induced complexity in the analysis, TRUEFAD was designed to only identify the main pure fiber types, type I, type IIA and type IIB + IIX. Another limitation is that TRUEFAD was developed under Windows. Although it should also work on Mac OS and Linux system, some macro error may arise. To limit this risk, we recommend to not use the batch mode that enable TRUEFAD to work in background.

With this publication, we hope to start an international work group to optimize the workflow developed here and be sure to help scientists all over the world to analyze their images related to muscle research with open-source pipelines adopted by users. Future versions and updates will be shared with the community, notably to optimize TRUEFAD on different operating systems. Current development is focused on the utilization of TRUEFAD Cells on low-resolution cameras as well as human myotubes. We can hypothesize that TRUEFAD could be adaptable to other types of cells or tissues, but these applications should be tested.

## Methods

### TRUEFAD cells—cell culture

C2C12 myoblasts were purchased from ATCC (Manassas, VA, USA) and maintained in Dulbecco minimum essential medium (DMEM) containing 4.5 g/L glucose (Sigma, L'Isle d'Abeau Chesnes, France), 10% fetal bovine serum, 100 UI/ml penicillin and 100 μg/ml streptomycin (PAA, Velizy-Villacoublay, France) at 37 °C in a 5% CO_2_ humidified atmosphere. When cells reached 80%–100% confluence, the medium was replaced with DMEM containing 4.5 g/L glucose, 2% heat-inactivated horse serum, 100 UI/ml penicillin and 100 μg/ml streptomycin to differentiate cells into myotubes^38^. For dexamethasone experiment, after 4 days of differentiation, cells were washed 3 times with phosphate-buffered saline (PBS) to reduce contaminating serum proteins and cell atrophy was then induced by dexamethasone (DEX, Sigma-Aldrich, Missouri, USA) at 10^–6^ M for 48 h. To assess the effects of IGF-I treatment on cell hypertrophy, differentiated cells were treated with LONG® R3 IGF-I human recombinant (50 ng/ml, Sigma-Aldrich, Missouri, USA) for 48 h in 2% HS differentiation medium.

Human skeletal muscle cells (SkMDC, Cat. #Sk-1111) from a 41-year-old donor were purchased from Cook MyoSite (Pittsburgh, PA, USA) and cultured in growth medium consisting of DMEM with GLUTAMAX ITM, 20% FBS, 0.5% Ultroser G (Pall, Cergy, France), 0.1% gentamycin and 1% nonessential amino acids at 37 °C in 5% CO2 incubator. Their mean population doubling level was determined at each passage. After 4 days of differentiation in DMEM supplemented with 1% FBS/2% HS, cells were treated with DEX at 10–6 M for 8 h.

### TRUEFAD Cells—manual myotube morphological analysis

Myotubes were photographed directly in culture plate without fixation using an AxioCam 305 color digital camera coupled to an AxioVert.A1 microscope with the use of ZEN 2.5 software (Zeiss, Germany). Myotube diameter was measured as previously described^34^. Briefly, diameters of 75 myotubes in each condition from 3 independent experiments were evaluated. For each myotube, three random measurements were performed along the length of the myotube (n = 5 measurements/myotube) using the ZEN 2.5 software, and the average of these three measurements was considered as one single value.

### TRUEFAD Cells—manual image annotation and training set

Over 300 images with a resolution of over 2000 × 2000 pixels were used to train our deep-learning model. Cells following various treatment, including IGF1 and Dexamethasone, were also required to be sure TRUEFAD Cells will still detect myotubes in challenging situations. Our annotation method for all images was to draw a yellow line in top of the grey scale image corresponding to the center of each myotube with an Ipad 2.0 pro among a total of 13 different experimenters. Three experts have checked individually 100 images to verify the correct detection of all myotubes and thus estimate the percentage of false positive labels. Once all the verification job was done, all overlays were obtained from respective images thanks to the color threshold function on FIJI and saved as binary 8-bit images for a total of 17,283 myotubes. All images were cropped and scaled down to 512 × 512 pixels to ensure efficient subsequent training on Google Collab.

### TRUEFAD Cells—deep learning model of myotube detection

TRUEFAD Cells workflow relies on a semantic deep learning model that was trained thanks to the ZeroCostDL4Mic Google Collab Notebook based on 2D-UNet architecture^39^. Deep learning “Myotube detection” model was trained from scratch on the annotation set previously described with a patch size of 512 × 512, corresponding to the image dimensions, a batch size of 4 and no data augmentation. The training loss is a binary crossentropy loss weighted to avoid class imbalance between the prominent “background” regions in the image compared to the smaller “myotube” regions. The U-Net parameters were updated with an Adam optimizer and a decreasing learning rate initialized at 0.0003 and subsequently reduced by a factor of 10 at 75% of the training and by a factor of 100 at 95% of the training. The U-Net model was configured with two pooling layers. The model's performance was monitored during training using a validation set that consisted of 10% of the 300 image-annotation pairs. 50 epochs with 68 training steps each were necessary to obtain adequate IoU. Training and validation curves can be seen on Extended Data Fig. 4.

Once the model was tested, it was exported and installed on FIJI thanks to DeepImageJ plugin^31^. All the datasets and models are available online (see Data and Code availability section). Quality control of the model can be found under the Extended Data Fig. 1 section: “Model prediction metrics”.

### TRUEFAD Cells—FIJI pipeline

TRUEFAD Cells requires the installation of a few dependencies to fully and automatically run on an image folder from FIJI^21^: MorphoLibJ^32^, DeepImageJ^31^ with “Myotube detection” model installed, and ReadAndWriteExcel. TRUEFAD Cells starts with a graphical user interface that asks the user to select putative myotube retention parameters (Extended Data Fig. 5a) as well as input and output directories. Default parameters have been empirically tested during development but the user can customize them in different ways. With the following step-by-step workflow description, we will explain these diverse parameters.

TRUEFAD Cells will open individually each 8-bit image from the input directory and will treat them with the same parameters. The image is firstly cropped to get a proper square corresponding to the minimum value between pixel width and pixel height. Parsing fusion issues were found with rectangle images so we decided to crop all images in the same way to limit the loss of development time. A copy of the image is then scaled down to 512 × 512 pixels and sent to the DeepImageJ “myotube detection” model treatment. Once the myotube prediction is obtained, it is scaled back to the original size of the cropped image and follows an adaptive closing directional processing (*type* = *Min operation* = *Closing line* = *10 direction* = *15*) as well as a directional median processing (*type* = *Min operation* = *Median line* = *5 direction* = *15*). Threshold (*0–30,000*) is constantly run on the 32-bit prediction allowing to get the highest probability of myotubes in the image. This Threshold serves as a frame to make a top-hat filter (*user-defined parameter; Default* = *10*) on top of putative myotubes from the original image. This is where, depending on the image acquisition and the size of the cell, the user might change the parameter for “Border siding the DL prediction” (*user-defined parameter; Default* = *5*). Selection is after that inverted to add artificial noise (user-defined parameter; *Default* = *15*) on other elements that do not correspond to putative myotubes. After these filters, the image is segmented using a marker control watershed based on extended minima imposition and connected component labeling (morphological segmentation algorithm). The user can change the extended minima tolerance at the beginning (*user-defined parameter; Default* = *35*). Resulting labels are trimmed by their size (*Default min* = *750 *µm^2^*; Default max* = *120,000 *µm^2^*;*), their proximity to the field border (*KillBorder function*), and finally by their Geodesic elongation (*Range set up by the user; Default min* = *6; Default max* = *30*). “True myotubes” are extracted one by one from the label map, are smoothed by a morphological closing filter and are exported as an ROI. Each of these myotubes is interpolated and measured in nine equidistant sections perpendicular to the main myotube axis (Extended Data Fig. 5b). Myotube diameter, as well as area and orientation, are exported for each myotube on the image via the ResultToExcel plugin while ROIs map is exported as TIFF with myotube final overlays on top of the original image in the Output directory.

### TRUEFAD Histo—histological samples preparation

All animal experimental procedures were performed in accordance with Clermont-Ferrand University (France) IRB and ARRIVE guidelines. The study was approved by the local ethics committee (permission number 10635-2017071711566890v2). 6-week-old male WISTAR rats (n = 30, Janvier-Labs) were single-housed under controlled conditions of lighting (12 h light, 12 h dark cycle) and temperature (22 ± 2 °C). After an acclimatization period, they were fed with a casein or a plant-based food diet for 28 days. At the end of the protocol, 16-h-fasted rats were weighed and anesthetized with isoflurane and Gastrocneniums (GA) muscles were harvested and weighed. For histological analysis, one of the two GA muscles was mounted with tissue freezing medium (OCT), frozen in isopentane cooled on liquid nitrogen, and stored at − 80 °C. Serial cross-Sects. (10 μm thick) were performed using a cryostat at − 20 °C.

### TRUEFAD Histo—manual fiber type and cross-sectional area

It is known that rodent muscles contain 4 types of fibers, type I, type IIA, type IIB and type IIX, although in human only type I, IIA and IIX fibers exist. These different types express different myosin heavy chain isoforms with distinct metabolic activities, oxidative for type I, glycolytic for type IIB and IIX, and both activities for type IIA. As for technical reasons and because our aim was to identify a shift between oxidative and glycolytic metabolisms, we did not distinguish between IIB and IIX fibers in TRUEFAD for the pipeline dedicated to the detection of type I and IIA fibers. Fiber type and cross-sectional area (CSA) were measured as previously described^16^. Briefly, cross-sections were co-labeled with monoclonal antibodies against myosin heavy chain-I (BA-F8, DSHB), myosin heavy chain-IIA (SC-71, DSHB) and with anti-laminin-α1 (Sigma, Saint-Quentin-Fallavier, France) to outline the fibers. The labels were resolved with corresponding secondary antibodies conjugated to Alexa-Fluor 350, 488 or 546 (Invitrogen, Cergy-Pontoise, France. Images were captured with AxioCam 305 color digital camera coupled to an AxioVert.A1 microscope at a resolution of 0.548 μm/pixel. Five fields, each containing 100 fibers, were analyzed per animal. The fiber type (I, IIA or IIB + IIX) and cross-sectional area (CSA) were manually determined for each fiber, using the image processing software ImageJ 1.47v. Fiber type IIB + IIX will be attributed at each fiber which don’t have any pure type I or type IIA label.

### TRUEFAD Histo—FIJI pipeline

TRUEFAD Histo relies on two dependencies installation to fully automatically run on an image folder from FIJI: MorphoLibJ and ReadAndWriteExcel. TRUEFAD Histo proposes four different pipelines that will be developed after (1/“Import and work on label image”, 2: “Segmentation of laminin image”, 3: “Type attribution Laminin + Type I + Type IIA”, 4: “Type attribution Laminin + Type I + Type IIA + Type IIB/IIX”)**.** As the mechanism for the third and fourth pipelines are quite similar, we will present them together.

- 1/Import and work on label image
  This pipeline should be use only on images already processed for segmentation and labeling. Once this pipeline is selected, four different choices are available: “Label edition”, “Remove a specific label or group of labels”, “Looking for a label”, “Labels to ROI”. These choices rely mainly on MorphoLibJ commands with few adaptations.
- 2/Segmentation of laminin image
  This pipeline segments fibers from a fluorescent laminin image, or dystrophin for the protocols using this antibody instead, it might be used for testing different parameters or simply to analyze fibers on one image without further type attribution.
- 3/Type attribution Laminin + Type I + Type IIA + (Type IIB + IIX)
  This is the main pipeline for image analysis proposed by TRUEFAD Histo allowing segmentation and analysis of fiber composition in the same run of analysis. The user is firstly asked to select the three different Input folders directory (1/Laminin, 2/Type I, 3/Type IIA) and the Output directory. The first graphical user interface (Extended Data Fig. 6) is explaining how type attribution is made and is asking if the user wants to change the upper and lower threshold for Type IIB + IIX attribution. A second one (Extended Data Fig. 6) allows users to select analysis parameters for batch processing. These parameters will be described during the next step-by-step pipeline description. When TRUEFAD Histo starts, it opens the first segmentation (using laminin in our example) image in the folder and will process it as such: Firstly, depending on user preferences—if the laminin signal is weak or heterogenous—a “Find Edges” filter might be used. Then, directional median processing is run over the laminin image (*user defined parameter; Default* = *20*) followed by an adaptive Enhance Local Contrast (CLAHE) filter (*see above parameters calculation*). This adaptive CLAHE filter is also run over Type I and Type IIA fluorescence and relies on mean image grey value and image resolution.
  $$\begin{gathered} {\text{CLAHE arguments: blocksize}} = {\text{X, histogram}} = 256,{\text{ maximum}} = {\text{Y mask}} = {^*\text{None}^*}''{\text{with}}: \hfill \\ X = Math.round\left( {ImageWidth/5} \right)\quad Y = \left( {MeanGreyvalue/60} \right)/0.05; \hfill \\ \end{gathered}$$

TRUEFAD Histo starts the segmentation of the laminin label (or any label allowing the outline of fibers) with a morphological segmentation algorithm. The user can change the extended minima tolerance at the beginning (*user defined parameter; Default* = *20*). Resulting labels are trimmed by their size (*Default min* = *2000 *µm^2^*; Default max* = *200,000 *µm^2^), their proximity to the field border (*KillBorder function*) and finally by their Geodesic elongation (*user defined parameter; Default max* = *4*). The resulting labels are eroded (*user defined parameter; Default* = *3 pixels).* At this step, the user might want to stop and check the label segmentation that has been done (*Manually edit labels post filtering checkbox at the beginning*). Labels are then transformed to ROIs and are placed over Type I and Type IIA fluorescence images to measure mean grey intensity for each fiber on each channel. Type attribution probability is calculated as such:

$$- 1 \le Type \, IIA < - 0.2 > Type \, IIB/IIX < + 0.2 < Type \, I \le + 1$$

Based on this calculation, hybrid fibers expressing both type I and IIA myosins would exhibit high grey values on both channel and then misclassified as IIB or IIX. Hence, if requested at step 3 of the pipeline, putative hybrid I/IIA fibers will be identified in the last column of the excel report as “Hybrid?”. In this case, fibers have a type I and type IIA signal, respectively higher and lower than the user-defined threshold at Step3 (Delta Comparison). All morphometry measurements (fiber surface, perimeter, shape) and type attribution-related information are exported in one Excel file.

### Quality control of the different analysis

All analyses shown on Figs. 2, 3, 5 with TRUEFAD Cells and TRUEFAD Histo were done using default user defined parameters. All analyses were run fully automatically on a Windows 10 Pro V.21H2 × 64 computer with i7-11850H 2.5 GHz/RAM: 32Go DDR5/FIJI: 1.53q. Interindividual measurement of five myotubes in Fig. 2d was made by 10 different experimenters while Figs. 2c,e, 5c,e quality control assessments were made by 3 different experimenters. Dice coefficient was calculated using MorphoLibJ label comparison between TRUEFAD and handmade label of the same selected myotube. Downscaled resolution tests made on TRUEFAD Histo on Fig. 5 were executed in 58.3 s/Image on 1.0X; 27.3 s/Image on 0.75X; 10 s/Image on 0.5X; 2.8 s/Image on 0.25X. Figure 2e Dice coefficient measurement was performed thanks to the MorpholibJ library.

### Statistical analysis

All data presented are mean ± standard error about the mean (SEM). For statistical tests, a Bartlet test prior to a one-way analysis of variance (ANOVA) was performed on Fig. 3b,c, followed by a Tukey post-hoc multiple comparison tests. Friedman test with Dunn’s correction was performed on Fig. 5c. Paired t test were used on Fig. 3d,e data. Pearson correlation between TRUEFAD results and manual results were performed on the same raw data in Figs. 2f, 5f. The number of experiments and samples was indicated in each legend of figures. All statistical analyses were performed using GraphPad Prism 9.5.0.730, November 9th, 2022 Version.

### Supplementary Information

Supplementary Information.

## Data Availability

Bioimage io model zoo: All training dataset and the myotube detection model are available at this address: https://github.com/AurBrun/TRUEFAD/releases/tag/data%26model. TRUEFAD Cells and Histo Output: All results for the different experiments are available in an Excel file.

## Code availability

GitHub repository: Both TRUEFAD Histo and TRUEFAD Cells complete code are available on GitHub from this link: https://github.com/AurBrun/TRUEFAD Other resources are also available for scientists that need help formatting their data, installing the package and dependencies with links to the Video Tutorial. This repository compiles information concerning the model training dataset. Video tutorial: A youtube Video tutorial is available and describes: 1/ TRUEFAD package objectives. 2/ TRUEFAD dependencies and package installation. 3/ How to run TRUEFAD Cells?. 4/ How to run TRUEFAD Histo?. 5/ How to deal with common issues?. 6/ How to deal with the data?. 7/ Get in touch with the authors.
