# Supplementary material for: A new bio imagery user-friendly tool for automatic morphometry measurement on muscle cell cultures and histological sections
Source: Sci Rep. 2024 Feb 7;14:3108. doi: 10.1038/s41598-024-53658-0 (PMC11269594; doi:10.1038/s41598-024-53658-0)
Supplement: Supplementary file 1 — Supplementary Information. [file 41598_2024_53658_MOESM1_ESM.docx]

**Supplementary information**


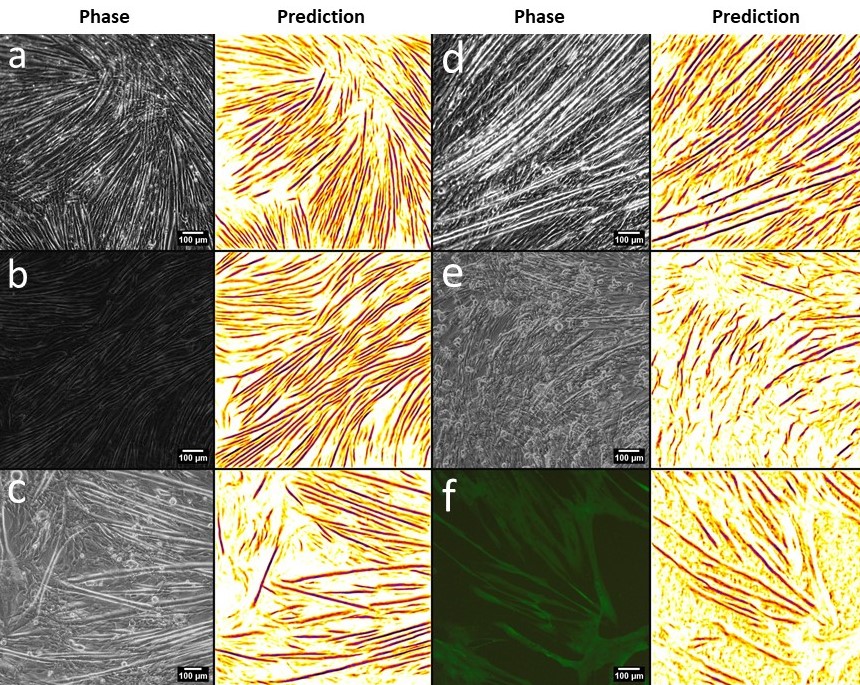


**Extended Data Figure 1**. Model prediction robustness.

Various 8-Bit original images obtained with different cameras and experimenters are shown from (**a**) to (**f**) left panels, meanwhile, the right panel shows their respective myotube probability prediction obtained after running TRUEFAD myotube detection model. The model was trained on 17,283 myotubes annotated by hand with 300 images that might be underexposed such as (**b**), slightly contrasted (**c**), or heavily contrasted (**d**). Predictions are still relevant when there is a low amount of myotubes following atrophy or toxic treatments (**e**)**.** However, our model shows limits on fluorescence images of human myotubes (**f**).

**
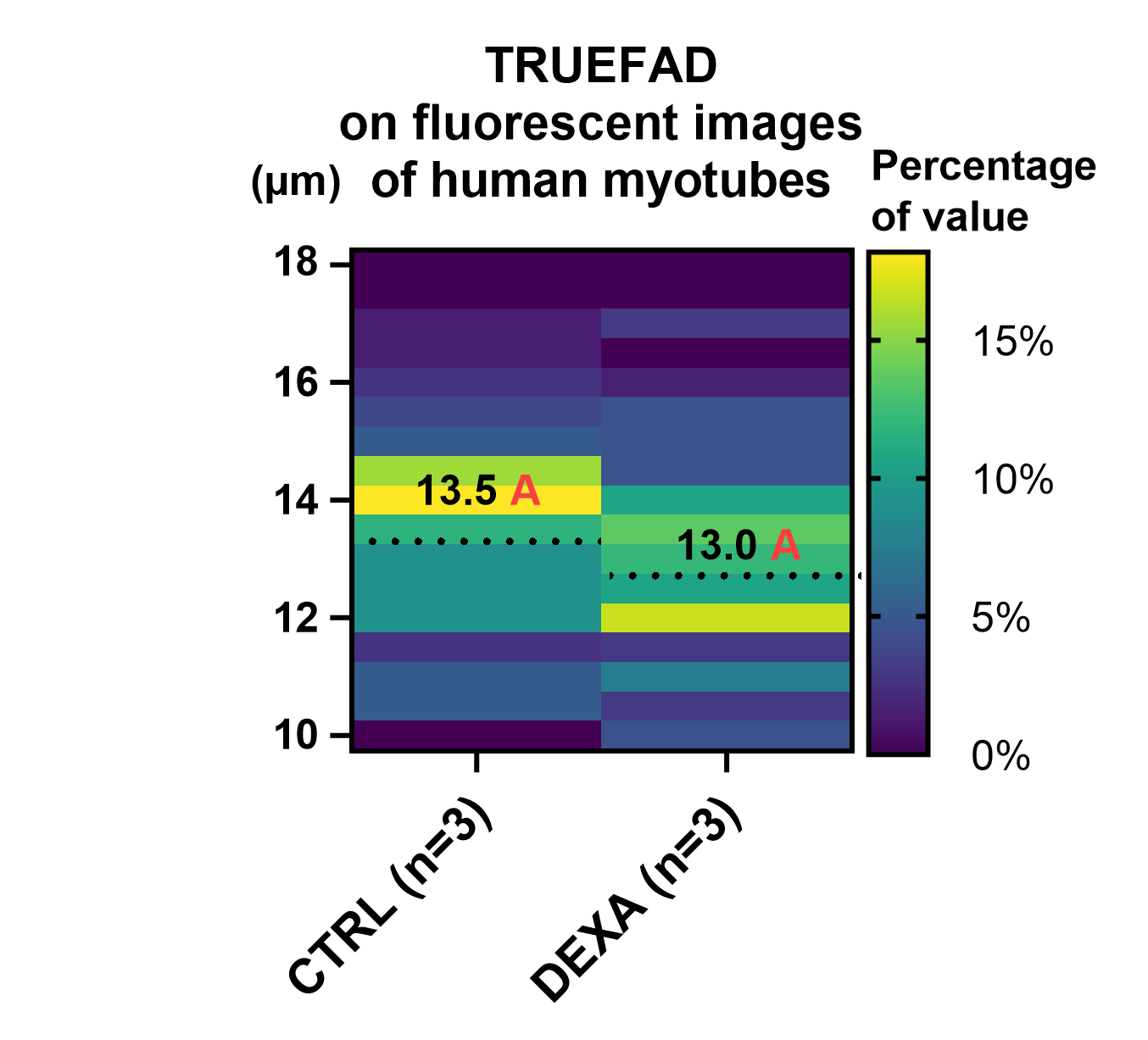
****Extended Data Figure 2.** TRUEFAD Cells working on fluorescent images of human myotubes. Prospective atrophy detection by TRUEFAD on human myotubes exposed to 48h of Dexamethasone (DEXA) treatment on n=3 different experiments (*13.45±0.16 vs. 12.99±0.22 ; p = 0.039 ; myotube nb. = 77 CTRL vs 66 DEXA*).

**Extended Data Figure 3.** Correlation between manual and TRUEFAD histo CSA measurements independently of the fiber type. Pearson correlation test developed by type in Fig. 5(**f**), were regrouped with each point corresponding to a single rat (sum of data coming from 5 to 7 different fields).
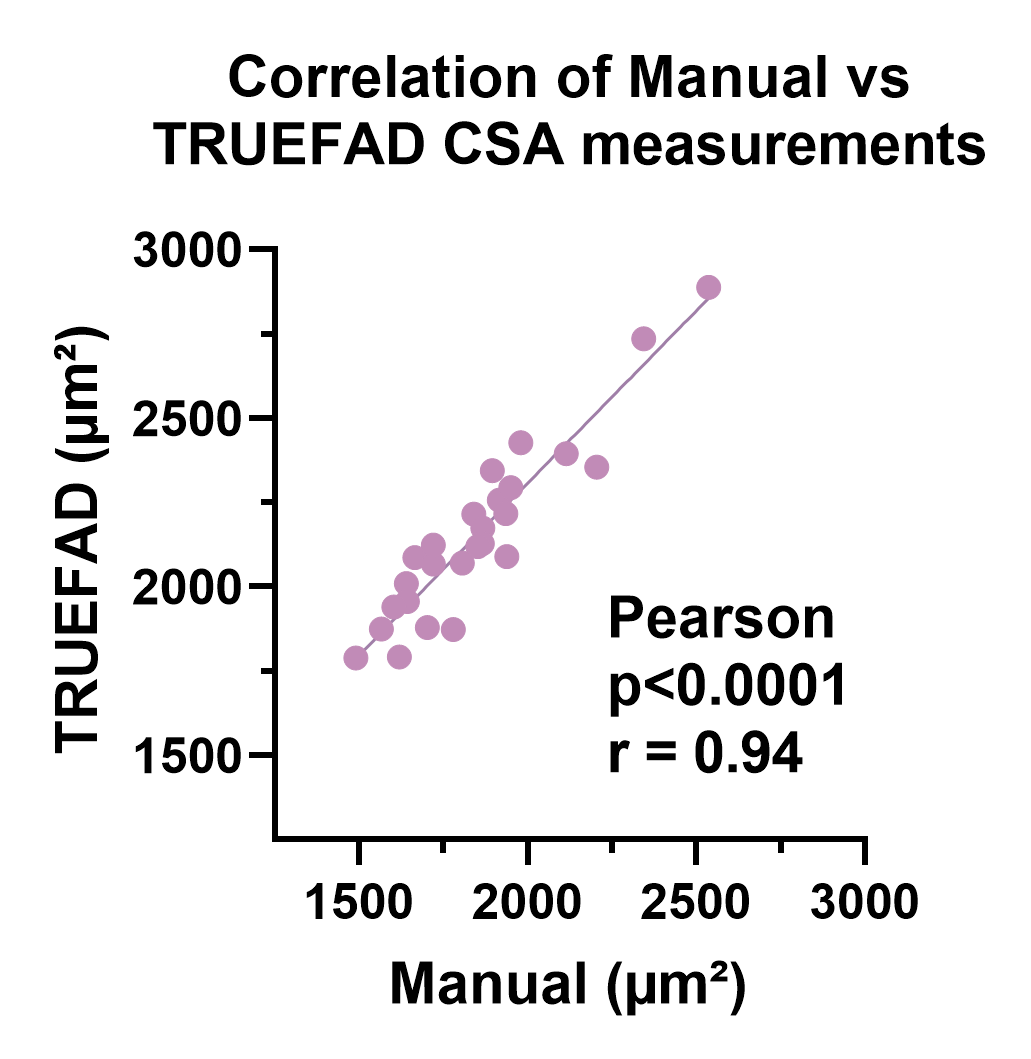


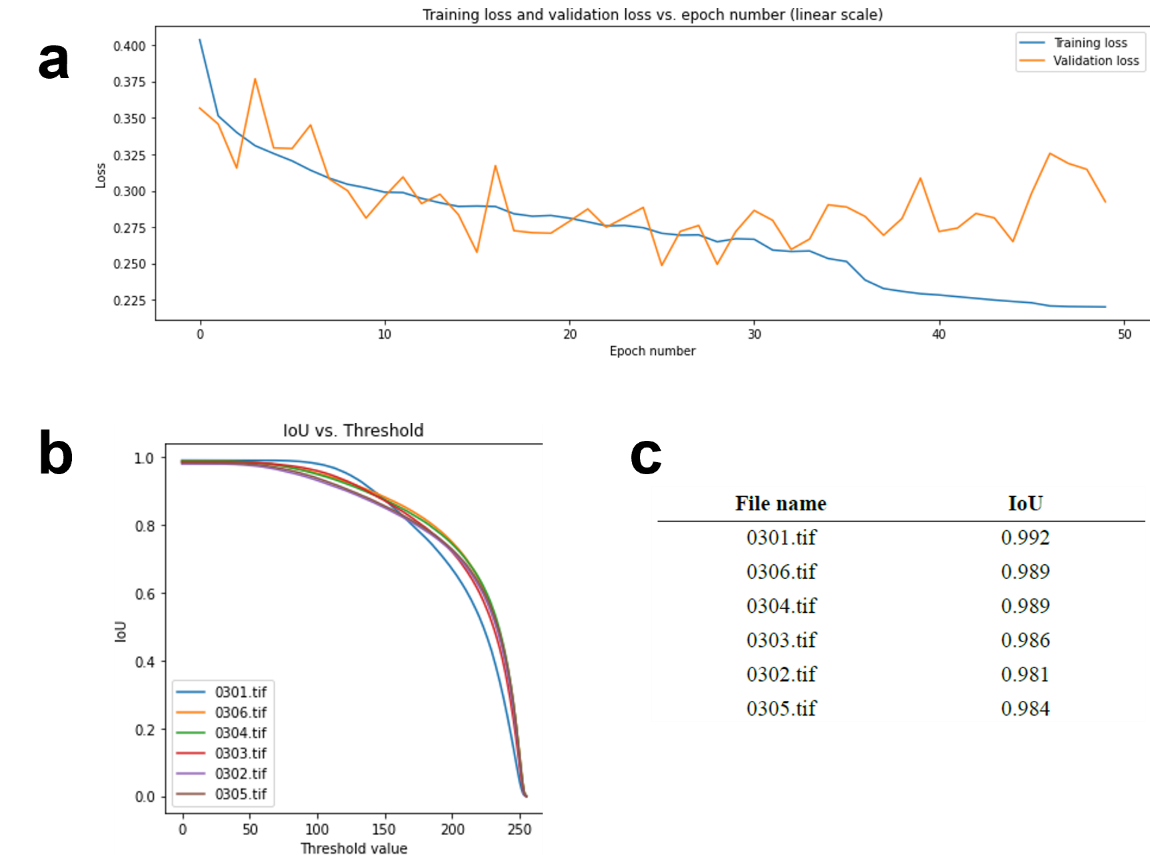


**Extended Data Figure 4.** Model prediction metrics.

(**a**) Training and validation curves obtained with ZeroCostDL4Mic notebook. The validation and training curves both decrease until the end of the training. This illustrates why the training has been stop at 50 epochs as a longer training would cause overfitting, meaning that the validation curve might re-increase after this period. (**b**) As the model raw output is a non-binary image, it must be thresholded. The best threshold is selected based on the performance of the model on the test set for different threshold values. (**c**) Performance of the model for the test images.

##
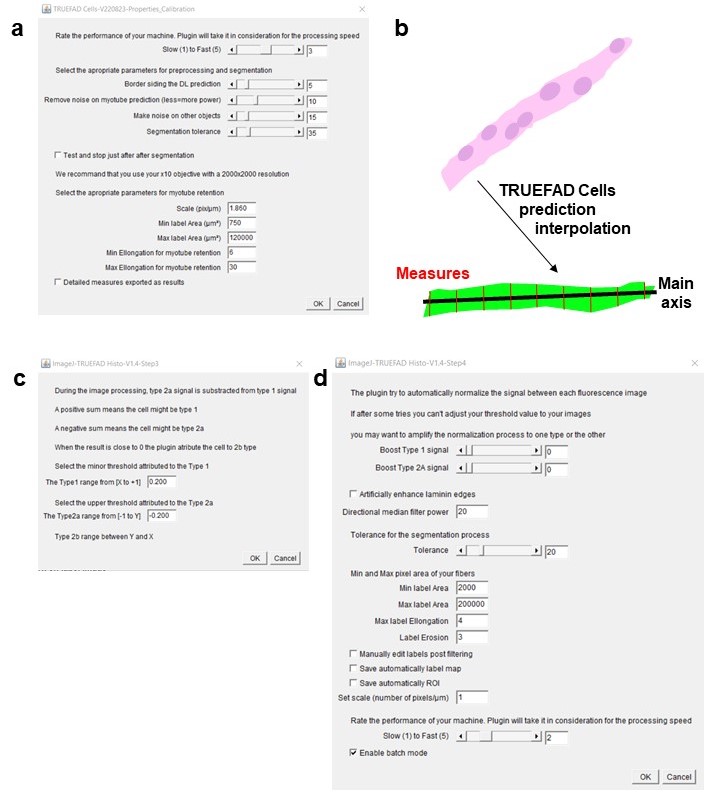


**Extended Data Figure 5**. TRUEFAD Cells workflow supplementary data. (**a**), TRUEFAD Cells graphical user interface at the start. (**b**), TRUEFAD Cells prediction interpolation and measurement along the main myotube axis.

For TRUEFAD histo pipeline, the first step is to create on your computer one folder for each label, depending on the type of analysis to be carried out (see choices below). For example, if laminin, and Type I and Type IIA fibers labeling are going to be analyzed, you need 3 distinct folders containing the corresponding images with exactly the same filename (ex « sample1.tif » in folder laminin, « sample1.tif » in folder Type I, and « sample1.tif » in folder Type IIA). Only image files to be analyzed should be found in folders. An additional empty folder must be created for result files. In the following procedure and current version of TRUEFAD, default labeling are laminin for fiber segmentation, BAF8 for type I and SC71 for type IIA labeling respectively (other labels are compatible and could be used instead).

- load TRUEFAD macro in Fiji

-Select « run »

- You will get a new window to inform that MorpholibJ and Read and Write Excel Package Plugins must be previously installed in Fiji.


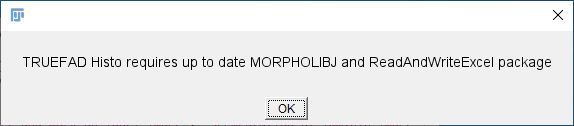
 click on « OK »

- You will get a new window :


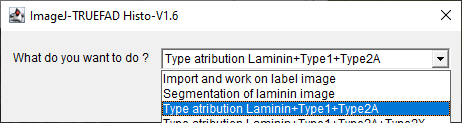


Four options are available, depending on the objective of the analysis (see the main manuscript for further details) :

- « Import and work on label image » to work on a previously processed label map.
- « Segmentation of laminin image » to segment fibers using laminin (or any label allowing the outline of fibers). In this case, only one folder is necessary.
- « Type attribution Laminin+Type1+Type2A ». Includes segmentation + labeling of type I and type IIA fibers (as explained in the manuscript).
- « Type attribution Laminin+Type1+Type2A(+Type2X). Includes segmentation + fiber typing using 3 or 4 labeling in the same pipeline.

-Click « ok ». You will get a new window:


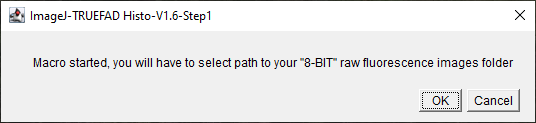


- Click « ok ». You will get successive new window to select the paths for the folders corresponding to each labeling, namely here laminin, BAF8 and SC71 following the order specified in the name of the windows.

-Once folder are identified, you will get a new window:


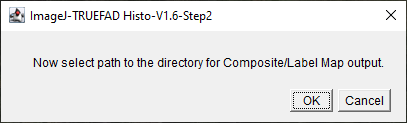


- Click « ok » and select the path for the result’s folder

- You will get a new window:


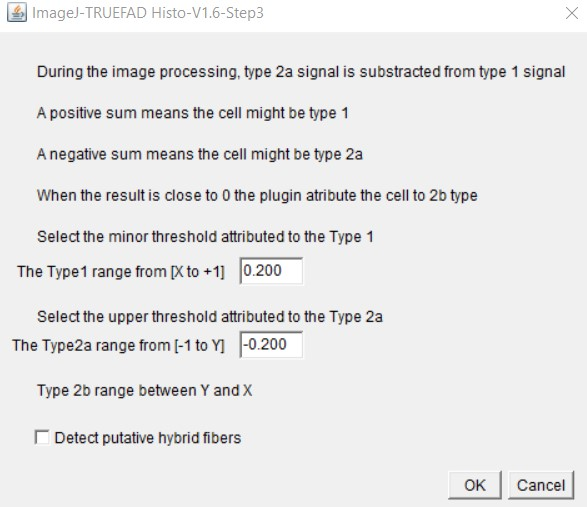


It is here possible to adjust the probability threshold to assign fiber to type I or IIA. We recommend to keep the default parameter but adjustments may be necessary. Few images from a batch may be firstly checked manually using a different threshold and rerun TRUEFAD. The user has the possibility to request for an automatic detection of putative hybrid fibers (a manual analysis of raw signal intensities (available in the excel file obtained at step 5) is however highly recommended).

Click « ok »

- You will get a new window to set parameters related to image quality, resolution and outputs:


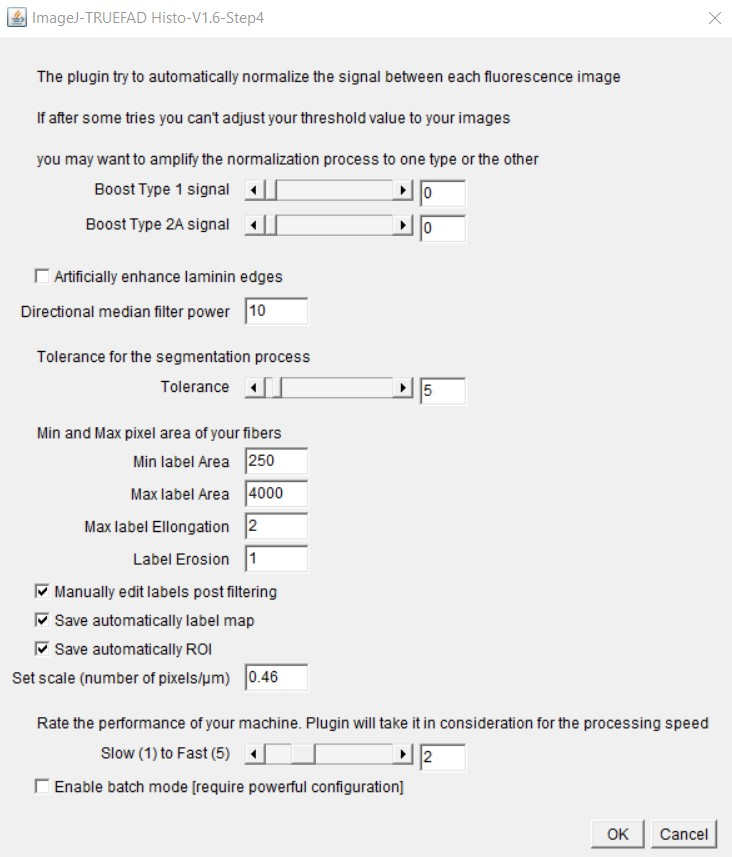


Check to enable working in background

To increase labeling signal

Increase the edge of the sections

Should be increased to remove noise, but reducing resolution

Change may be necessary to improve segmentation

Select filtering according to fiber size or shape

You can choose to save different processed pictures, remove or fuse labels

Set to your image resolution

To adapt TRUEFAD to your computer ressources

It is important to note that working in batch mode may led to the interruption of the process as depending of the resources of the computer, some bugs may occur. We recommend to keep this option disabled.

Click « ok »

- You will get a new window then click« ok ». The analysis is now running.


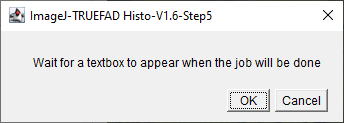


-At the end of the analysis, few windows open, please click « ok » for each.

- Results (label map, image composite, ROI listing) could be retrieved in the path selected by the user. A detailed quantified output could be found as an excel file « rename we after writing is done saved on the computer desktop ».

-We recommend to check all images for consistency in segmentation and fiber type identification, notably for hybrid fibers. If not, rerun TRUEFAD with different settings.

**Extended Data Figure 6. Step by step protocol for TRUEFAD histo**
